# P28 Immune reconstitution-induced sarcoidosis following initiation of antiretroviral therapy in an HIV-positive patient

**DOI:** 10.1093/rap/rkae117.059

**Published:** 2024-11-05

**Authors:** Hlaing Myat Chit Su, Joel Conway, Simran Grewal

**Affiliations:** Barts Health NHS Trust, London, United Kingdom; Barts Health NHS Trust, London, United Kingdom; Barts Health NHS Trust, London, United Kingdom

## Abstract

**Introduction:**

Sarcoidosis is a multi-system granulomatous disease which is histologically characterised by multiple non-necrotising granulomas mainly composed of CD4(+) and CD8(+) lymphocytes. Human immunodeficiency virus (HIV) infection is well known to cause reduced CD4(+) T cell count, causing impaired immune system and further developing into acquired immune deficiency syndrome (AIDS). Thus, the coexistence of HIV infection and sarcoidosis is extremely rare. We report a case of a 39-year-old HIV-positive patient who has developed sarcoidosis in his skin and lymphatic system following highly active anti-retroviral therapy treatment (HAART).

**Case description:**

We present a case of sarcoidosis in a 39-year-old male with well-controlled HIV, who presented with incidental splenic lesions and multiple lymph node and lung nodule uptakes on CT PET scan. His medical history included HIV, syphilis, NAFLD, hypothyroidism, suspected vitiligo, psychosis, and exposure to a CCR5 receptor antagonist through the HEPMARC clinical trial. He had been prescribed tenofovir 245 mg / emtricitabine 200 mg and raltegravir 1200 mg OD for three years prior to the onset of sarcoidosis. The patient reported occasional non-exertional shortness of breath as his only notable symptom. Clinical examination revealed pigmented skin lesions and palpable lymph nodes.

Blood panels at diagnosis included a white cell count of 4.3 x10^9/L (4-10); C-reactive protein of 3 mg/L (<5), serum angiotensin converting enzyme level of 147 U/L (20-70). His CD4 count was 509 x10^6/L (455-1320) in the month of the lymph node biopsy and his HIV1 RNA level has been <50 since diagnosis in 2021.

A broad autoimmune panel had no significant positives. A groin lymph node biopsy was performed which demonstrated multiple lymph node fragments showing almost total replacement of the lymph node by scattered non-necrotising granulomas.

A Ziehl-Neelsen and Wade-Fite stains for acid fast bacilli and PASD and Grocott stains for Fungi were all negative. An immunostain for treponema was also negative. Additional supportive diagnostic measures included skin biopsy and bronchoscopy.

Punch biopsies of the pigmented skin lesions demonstrated numerous confluent non-caseating granulomas of sarcoidal type, composed of epithelioid histiocytes and multinucleated giant cells, occupying the whole thickness of the dermis. Occasional asteroid bodies were seen.

Bronchoscopy direct spreads were cellular and showed numerous mature lymphocytes and occasional epithelioid histiocytes, forming non-caseating granulomas. Initial management consisted of a prednisolone weaning course starting at 20 mg.

**Discussion:**

HIV infection reduces CD4(+) T cell count, the main component cells of sarcoid granuloma formation. It is extremely rare to see both conditions coexisting due to the paradoxical roles of CD4(+) T cells in the pathogenesis of sarcoidosis and HIV infection. Currently, there are 55 cases reported worldwide on concomitant sarcoid and HIV infection, of which 14 were pre-HAART era. The peripheral CD4(+) cell count were above 200 x10^6/L in most patients who have developed sarcoidosis as in our case.

‘Immune reconstitution’ of HIV infection after HAART is thought to play a role in the pathogenesis of sarcoidosis in HIV-positive patients. It is characterised by clinical manifestations of inflammatory disorders or opportunistic infections and low pre-treatment CD4(+) T cell count, typically a few months after starting HAART. Our patient, however, developed sarcoidosis after 10 years of stable disease, although his pre-treatment CD4(+) T cell count was low (211x10^6/L) and viral load improved with treatment.

The temporal relationship between HAART and sarcoidosis is not typical for other immune-reconstitution syndromes. The naïve cells or IL 2 receptors positive CD4(+) T cells have more delayed recovery time (3-6 months) compared to the T memory cells (three weeks). The former plays the main role in sarcoidosis, whilst the latter for other immune-reconstitution diseases such as *Mycobacterium avium* complex. In this case, it has been 13 years post-diagnosis and treatment. Thus, it is assumed that the immune system has restored and causes development of sarcoidosis.

Sarcoidosis should be the diagnosis of exclusion in HIV-positive patients. Non-Hodgkin’s lymphoma, the most common type of lymphoma in HIV-positive patients, should be excluded. Endobronchial ultrasound (EBUS) guided lymph node biopsy significantly improves the diagnostic yield up to over 90% in suspected sarcoidosis, which also confirmed the diagnosis in this case.

**Key learning points:**

• In conclusion, it is important to recognise that sarcoidosis can develop in patients with HIV infection, especially after being treated with HAART. It can either happen within a few months, which is due to immune-reconstitution, or can happen after several years once the treatment has restored the normal immune system as in this case.

• It is, however, crucial that the diagnosis should only be made after careful exclusion of lymphoma and all the other opportunistic infections which can mimic sarcoidosis, as the treatment with immunosuppressives can otherwise worsen the latter.

• Clinicians should not disregard a possible diagnosis of sarcoidosis in patients with HIV infection considering the paradoxical roles of CD4(+) T cells in the pathogenesis. In fact, in current practice, as more patients with HIV have been diagnosed and treated quickly, better responses to such treatment have led to ‘almost normal’ immune response. As a result, the hyperinflammatory and autoimmune conditions can present also in HIV positive patients. Therefore, it is essential to think of and consider such conditions including sarcoidosis as a possible diagnosis in clinically suggestive cases.

• It is interesting to note that management of sarcoidosis is similar in both HIV positive and HIV negative patients. Many patients respond to prednisolone as in this case. In severe cases, more aggressive treatment with disease modifying agents, such as methotrexate, or biologic treatment, such as rituximab, might be considered. It is currently recommended that these can be started as long as the CD4(+) T cell count is over 200 x10^6/L and the HIV viral activity has been completely suppressed. However, prophylactic treatment should be given to all patients with HIV infection who have been started on immunosuppressive therapy.

